# Under‐ice mesocosms reveal the primacy of light but the importance of zooplankton in winter phytoplankton dynamics

**DOI:** 10.1002/lno.11618

**Published:** 2020-10-04

**Authors:** Allison R. Hrycik, Jason D. Stockwell

**Affiliations:** ^1^ Rubenstein Ecosystem Science Laboratory University of Vermont Burlington Vermont USA; ^2^ Biology Department University of Vermont Burlington Vermont USA

## Abstract

Factors that regulate planktonic communities under lake ice may be vastly different from those during the open‐water season. Expected changes in light availability, ice cover, and snowfall associated with climate change have accelerated the need to understand food web processes under ice. We hypothesized that light limitation (bottom‐up control) outweighs zooplankton grazing (top‐down control) influence on phytoplankton biovolume and community structure under ice in a north temperate lake. Using in situ under‐ice mesocosm experiments, we found that light had stronger effects on phytoplankton abundance than zooplankton, as expected. Specifically, low light limited growth of diatoms, cryptophytes, and chrysophytes. Zooplankton, however, also significantly affected some individual phytoplankton groups by decreasing diatoms and cryptophytes, in contrast to the common assumption that zooplankton grazing has negligible effects under ice. Ammonium and soluble reactive phosphorus (SRP) were lowest in high light treatments presumably through uptake by phytoplankton, whereas ammonium and SRP were highest in high zooplankton treatments, likely a result of zooplankton excretion. In situ experimental studies are commonly applied to understand food web dynamics in open‐water conditions, but are extremely rare under ice. Our results suggest that changes in the light environment under ice have significant, rapid effects on phytoplankton growth and community structure and that zooplankton may play a more active role in winter food webs than previously thought. Changes in snow and ice dynamics associated with climate change may alter the light environment in ice‐covered systems and significantly influence community structure.

The relatively recent and rapid changes in winter conditions in temperate zones have led to declining ice cover in temperate lakes (Sharma et al. 2019) and altered snow cover and snowmelt dynamics (Musselman et al. 2017). However, the impact of such changes in winter conditions on lake food web dynamics under ice is poorly understood (Salonen et al. 2009; Sommer et al. 2012) because winter limnology has been understudied compared to open‐water limnology (Salonen et al. 2009; Hampton et al. 2015). Experimental studies crucial to understand lake processes during the open‐water season (e.g., Schindler 1977) are missing during the ice‐covered season, despite the potential for winter plankton community dynamics to set inoculum conditions for the open‐water season (Feuchtmayr et al. 2012). For example, phytoplankton can reach bloom densities under ice (Katz et al. 2015) and support abundant zooplankton populations (Grosbois and Rautio 2018). A study of 101 lakes worldwide found that winter chlorophyll *a* (Chl *a*; a proxy for phytoplankton abundance) reached an average of 43% of summer Chl *a* (Hampton et al. 2017). To better understand if and how changes in snow and ice cover will affect biotic communities under ice and potentially into the open‐water season, we must first disentangle the drivers of food web dynamics under ice.

Physical factors such as light and temperature are considered the main drivers of winter phytoplankton biovolume in the Plankton Ecology Group (PEG) Model that describes planktonic community succession in temperate lakes (Sommer et al. 1986, 2012). Light limits photosynthetic activity and can be highly variable depending on winter conditions (Salonen et al. 2009; Hampton et al. 2015), including day length, ice thickness, ice clarity, and especially snow cover (Bolsenga and Vanderploeg 1992). For example, clear ice may allow greater than 70% transmittance of photosynthetically active radiation (PAR), while white ice without snow decreases PAR transmission to 15–31%, and snow on ice decreases PAR transmission to less than 20% (Bolsenga and Vanderploeg 1992). Predictably, total phytoplankton production is highest when light transmission is highest during winter (Maeda and Ichimura 1973). Additionally, community structure may be highly sensitive to changes in light levels because different taxa may be better equipped to deal with different light conditions during winter. For example, diatoms have been found at bloom densities in conditions with clear ice and minimal snowpack (Katz et al. 2015). Some phytoplankton taxa with adaptations that allow them to succeed during light‐limited conditions, such as mixotrophic or mobile flagellated taxa, are often found in high proportions under ice (Özkundakci et al. 2016). To this end, we may expect a higher proportion of known mixotrophic taxa, such as chrysophytes (Sanders et al. 1990), when light is limited, and higher total phytoplankton biovolume with high light transmission.

Zooplankton can control phytoplankton biovolume and community structure during the open‐water season through grazing, including selective feeding on specific phytoplankton groups (Bergquist et al. 1985). However, less is known about top‐down effects of zooplankton under ice, which makes interpretation and prediction of food web interactions under ice and entering the spring phytoplankton bloom difficult (Sommer et al. 2012). Zooplankton actively feed under the ice (Vanderploeg et al. 1992; Grosbois and Rautio 2018), although they may be heavily dependent on accumulated lipid stores (Grosbois et al. 2017). Similar to the open‐water season, zooplankton grazing rates and phytoplankton response during winter are expected to be dependent on the zooplankton and phytoplankton species present, and their interactions. For example, winter zooplankton communities are often dominated by copepods and rotifers (Blank et al. 2009). A higher ratio of herbivorous to predatory rotifers may be expected under ice if *Daphnia* are limited (Obertegger et al. 2011). Copepods may have particularly strong impacts on phytoplankton community structure through selective raptorial feeding (Sommer et al. 2003), suggesting that winter zooplankton communities that are actively feeding may influence under‐ice phytoplankton community structure and biovolume. Abundance of crustacean zooplankton may be highly variable in winter. Zooplankton density under ice may vary by several orders of magnitude across ice‐covered lakes (Hampton et al. 2017) and winter zooplankton biomass varies by more than one order of magnitude among years in our study system (A. R. Hrycik unpubl.).

Changes in nutrient concentrations under ice may be closely linked to changes in phytoplankton and zooplankton communities. Nutrients are generally not expected to limit phytoplankton growth during winter, especially in eutrophic systems (Sommer et al. 2012). However, we may still expect changes in nutrient concentrations resulting from phytoplankton uptake under ice. Higher phytoplankton growth generally corresponds with reductions in forms of nitrogen and phosphorus that can be assimilated quickly, such as soluble reactive phosphorus (SRP), ammonium, and sometimes nitrate (Glibert et al. 2016). Manipulation of zooplankton biomass may also alter nutrient levels through excretion (Oliver et al. 2015). Other sources of nutrient inputs that are important in the open‐water season, including phosphorus release from sediment (Penn et al. 2000), may also be a significant source of phosphorus under ice if oxygen is limited (Joung et al. 2017). We expected that nutrient concentrations would respond to changes in plankton communities but that nutrients would not limit phytoplankton growth (Sommer et al. 2012).

In this study, we used an in situ under‐ice carboy experiment to test the relative importance of zooplankton grazing vs. light limitation on winter phytoplankton biovolume, community structure, and nutrient concentrations in a north temperate lake. We hypothesized that under ice both low light and high zooplankton grazing would decrease phytoplankton biovolume and impact phytoplankton community structure, but that light would be quantitatively more important than zooplankton. Our hypothesis follows the PEG Model (Sommer et al. 1986) and its recent update (Sommer et al. 2012), in which physical factors are thought to shape winter phytoplankton communities compared to the higher influence of zooplankton grazing and nutrient limitation during the open‐water season. Our study manipulated only large‐bodied crustacean zooplankton to quickly alter grazing rate of a community without artificially altering phytoplankton communities (i.e., removal of small zooplankton would have also excluded colonial phytoplankton). To our knowledge, our under‐ice carboy experiment is the first application of this type of mesocosm experiment under ice, despite wide application of carboy experiments during the open‐water season.

## Methods

**Fig. 1:**
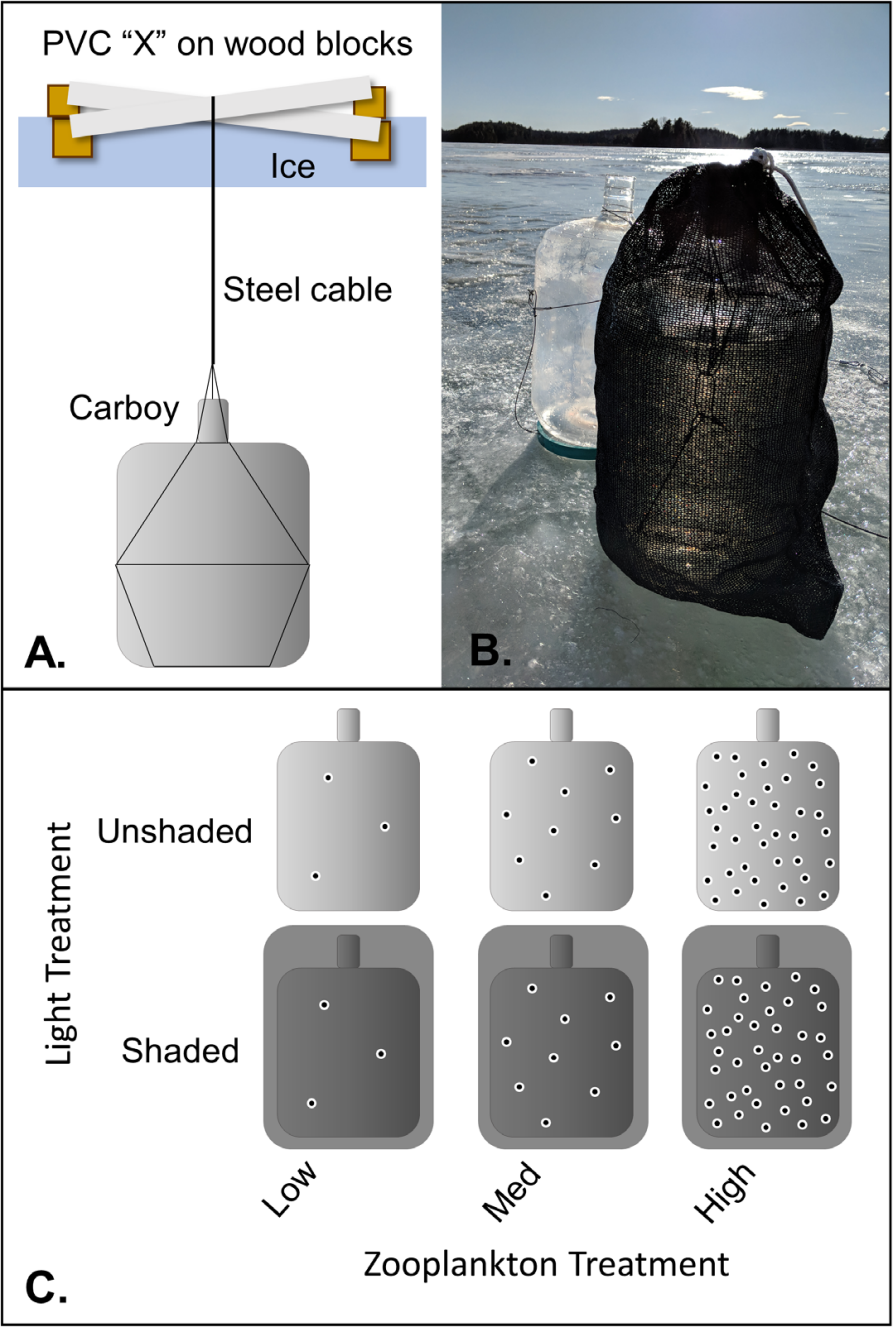
Carboy experimental design. (**A**) Each carboy was suspended in a randomized grid approximately 0.5 m below the ice by a steel cable harness connected to a PVC anchor in the shape of an “X.” PVC was placed on loose wood blocks to prevent it from freezing into the ice. (**B**) Greenhouse shade cloth covers blocked 85% of incoming light to simulate snow cover (photo credit: Hannah Lachance). (**C**) We crossed two light levels (unshaded and shaded) with three zooplankton levels with four replicates per treatment (*see* “Results” section for zooplankton abundances).

The experiment took place in Shelburne Pond, Vermont, a small, hypereutrophic system with a mean depth of 3.4 m and maximum depth of 7.6 m (Vermont Department of Environmental Conservation 2018). We initiated the experiment over two consecutive days with 12 carboys on 25 January 2018 and 12 carboys on 26 January 2018; we deployed two replicates of each treatment on each day. The transparent carboys were deployed on the north end of Shelburne Pond (44.39388°N, 73.16278°W) in an area with 0–2 cm of patchy snow on top of 30 cm of secondary ice (also called black ice; Block et al. 2019) and a water column depth of 4.6 m. We set carboys in a randomized grid pattern of six carboys by four carboys spaced 5 m apart. Each carboy was suspended by steel cable approximately 50 cm below the ice (Fig. 1A).

We tested four replicates for each of six treatments: (1) low zooplankton/unshaded, (2) low zooplankton/shaded, (3) medium zooplankton/unshaded, (4) medium zooplankton/shaded, (5) high zooplankton/unshaded, and (6) high zooplankton/shaded (Fig. 1B,C). We mixed ambient water for all treatments in a 208‐liter plastic barrel. The barrel was filled by raising and lowering the intake of a hand pump throughout the top 4.0 m of the water column and filtering through a 350‐*μ*m sieve to remove large zooplankton. Pilot work indicated 350‐*μ*m was the best mesh size to remove large grazing zooplankton but kept most colonial phytoplankton. The sieved ambient lake water in the barrel was mixed constantly as each group of six 22.7‐liter carboys (i.e., one replicate for each treatment) was filled.

We sampled phytoplankton, zooplankton, and nutrients while filling carboys to measure initial experimental conditions. We collected three 100‐mL water samples and preserved them in 1% Lugol's solution for phytoplankton enumeration. Three replicate 10 L water samples from the barrel that was previously sieved with 350‐*μ*m mesh were filtered through a 20‐*μ*m sieve for microzooplankton and rotifer enumeration. We also preserved the zooplankton filtered out of each barrel with the 350‐*μ*m sieve. All zooplankton and rotifers were anesthetized with Alka Seltzer before preservation with 70% ethanol. Finally, three replicate 500‐mL water samples were collected from each barrel for nutrient analyses. Once back at the lab, each nutrient sample was split into several different portions for nutrient analyses. A 100‐mL sample was preserved with three drops of sulfuric acid to achieve a pH of 2.0 for later analysis of total phosphorus (TP), total nitrogen (TN), and total organic carbon (TOC). Two 45‐mL samples were filtered through 0.45‐*μ*m syringe filters and frozen: one for SRP, and one for ammonia and nitrate + nitrite (NO_x_) quantification.

We then added zooplankton treatments directly to carboys. Two of the six carboys had no zooplankton added, two had ambient zooplankton added, and two had 10X natural abundance of large (i.e., sieved) zooplankton added. Zooplankton for the treatments were collected with a 13 cm diameter, 64‐*μ*m Wisconsin net through the upper 4.0 m of the water column and then retained on a 350‐*μ*m sieve. Desired densities were achieved for the medium zooplankton treatment to mimic ambient conditions by using a plankton splitter and adding half of the sieved net haul to each of the two ambient abundance treatments because half of the volume strained for a 4 m tow was approximately equal to the carboy volume. The high zooplankton treatments had sieved zooplankton from five zooplankton net tows added to each carboy. Finally, we covered one carboy from each zooplankton treatment with greenhouse shade cloth that blocked 85% of incoming light to simulate the light‐limiting effects of snow cover. This decrease in light transmission approximately corresponds to the difference between clear ice and combination (white and clear) ice with 3 cm of snow (Bolsenga and Vanderploeg 1992). The setup process was repeated twice on 25 January and twice on 26 January for a total of four replicates per treatment. All setup processes, including filtering zooplankton, were performed in the field at the time of deployment. We affixed a MK‐9 light and temperature sensor (Wildlife Computers, Redmond, Washington, U.S.A.) to the outside of one carboy without shade cloth and between another carboy and its shade cloth on 25 January. We also added a HOBO temperature sensor (Onset Computer Corporation, Bourne, Massachusetts, U.S.A.) to the inside of one shaded and one unshaded carboy on 26 January. Light values from MK‐9 sensors were converted from relative units to *μ*E m^−2^ s^−1^ (Kotwicki et al. 2009). We did not leave any head space in the carboys to simulate the sealed conditions of an ice‐covered lake. The ice over the carboys refroze within 1 d of deployment.

Carboys were extracted from the lake 14 d after deployment. Although most summer carboy experiments are much shorter in duration (e.g., Griniene et al. 2016), we expected that slow phytoplankton growth rates at low temperatures (Cloern 1977) would necessitate a longer incubation time. For example, *Cryptomonas*, which is common in Shelburne Pond during winter, has population growth rates of 6–7 times higher at 19–25°C than at 5°C (Ojala 1991). Upon retrieval, each carboy was inverted several times to homogenize contents before opening. We sampled phytoplankton, nutrients, and zooplankton from each carboy. We collected three phytoplankton samples and one combined nutrient sample (TP, TN, TOC, SRP, and NO_x_) using the same methods described above for initial sampling. We strained the remaining 21.84 L of water through a 20‐*μ*m Wisconsin net to sample crustacean zooplankton and rotifers. All zooplankton and rotifers were anesthetized with Alka Seltzer before preservation with 70% ethanol.

We identified phytoplankton to genus and counted full fields of view at 400x until reaching at least 300 natural units (cells for single‐celled species or colonies for colonial species). We measured 10 natural units per genus for each sample to calculate biovolume using Spot Basic software (Spot Imaging, Sterling Heights, Michigan, U.S.A.). Dimensions measured were dependent on phytoplankton taxa present, for example, we measured diameter for spherical cells and diameter and length for ellipsoid cells (Hillebrand et al. 1999). Within colonies, we measured 10 individual cells per colony when possible. If fewer than 10 cells were present or clearly visible, we measured all cells. We used those median measurements to calculate taxon‐specific biovolume for each sample (Hillebrand et al. 1999); then taxon‐specific biovolume was multiplied by cell abundance to estimate total biovolume per sample for each taxon. We only processed one out of the three phytoplankton samples collected per carboy because replicates within each carboy were very similar and would not have added to statistical power due to pseudoreplication. Analysis of three pairs of phytoplankton samples from the same carboys showed an average of 4.2% difference in cell counts for each genus.

We processed rotifers and crustacean zooplankton by measuring and counting at least 200 individuals per sample (200 rotifers and 200 zooplankton). Rotifers were counted and measured using a Nikon Eclipse Ni‐U compound microscope (Nikon, Tokyo, Japan) with Spot Basic software (Spot Imaging), while zooplankton were identified and enumerated on an Olympus SZX12 dissecting microscope (Olympus Corporation, Tokyo, Japan) interfaced with a GTCO CalComp digitizer for measurements (Turning Technologies, Youngstown, Ohio, U.S.A.). Rotifer and crustacean zooplankton biomass were calculated using length‐to‐mass conversions (all crustacean zooplankton and most rotifers) or length/width‐to‐mass conversions (*Filinia* rotifers) (Watkins et al. 2011; United States Environmental Protection Agency 2016).

We measured nutrient concentrations primarily to ensure that we did not artificially limit nutrients in our study. Nutrient samples were either stored frozen (SRP, ammonium, and NO_x_) or acidified and refrigerated (TN, TOC, and TP) until analysis. We measured TN and TOC on a TOC‐L total organic carbon analyzer with a TNM‐L TN measuring unit (Shimadzu Corporation, Kyoto, Japan). We analyzed TP and SRP using the molybdenum colorimetry method (USEPA 1993) with ascorbic acid modification and a persulfate digestion for TP on a Shimadzu UV‐VIS 2600 spectrophotometer (Shimadzu Corporation). Ammonium and NO_x_ were measured on a SEAL AA3 continuous flow autoanalyzer (SEAL Analytical, Mequon, Wisconsin, U.S.A.) using Method No. G‐171‐96 Rev. 15 with salicylate for ammonium and Method No. G‐172‐96 Rev. 18 for NO_x_.

All response variables (phytoplankton abundance and biovolume, rotifer abundance and biomass, and nutrient concentrations) were checked for normality using the Shapiro‐Wilk test (Shapiro and Wilk 1965) and for homogeneity of variance using Levene's test (Levene 1960). When either of these tests were significant, we transformed data to improve normality or homogeneity of variance. Response variables were then analyzed using two‐way ANOVA (*α* = 0.05) with zooplankton and light as predictor variables. ANOVAs used Type I sum of squares because sample sizes were balanced between factor levels. We then used the ANOVA output for variance partitioning to quantify the contribution (partial *R* ^2^) of zooplankton and light separately on response variables as well as the contribution of an interaction term between light and zooplankton. The values are reported as *R* ^2^_zoop_, *R* ^2^_light_, and *R* ^2^_light:zoop_. When ANOVA showed significant differences between zooplankton treatments, we performed Tukey's test (Tukey 1949) to examine pairwise differences between zooplankton treatment levels.

We performed nonmetric multidimensional scaling (nMDS) with Bray‐Curtis distance on phytoplankton species composition to visualize differences in phytoplankton communities between treatments using the R package “vegan” (version 2.4.3; Oksanen et al. 2019). We removed the small number (12%) of phytoplankton records where phytoplankton could not be identified to genus, which should not significantly alter nMDS interpretation (Pos et al. 2014). We used permutational multivariate analysis of variance (perMANOVA) with Bray‐Curtis distance and 999 permutations to test significance of light and zooplankton treatments on overall phytoplankton community composition (Anderson 2001; Oksanen et al. 2019).

## Results

Light and temperature behaved as expected throughout the experiments. Light was reduced by greenhouse shade cloth (Supporting Information Fig. S1). Temperature remained consistent between light and shaded treatments, although internal carboy temperatures were slightly higher than external water temperatures. However, the difference between internal and external temperature (< 0.3°C) was small compared to the overall increase in water temperature over the course of the experiment (Supporting Information Fig. S1).

Several response variables were transformed prior to ANOVA to improve normality or homogeneity of variance. Although most variables passed Shapiro‐Wilk and Levene's tests before or after transformation (Table 1), some variables had significant *p* values (at *α* = 0.05) for the Shapiro‐Wilk test (*p* = 0.02 for diatom abundance, *p* = 0.04 for cryptophyte biovolume, *p* = 0.03 for chrysophyte biovolume, *p* = 0.007 for chrysophyte density, and *p* = 0.006 for TOC) and Levene's test (*p* = 0.04 for haptophyte biovolume). We chose to continue with ANOVA because ANOVA tends to be robust to minor violations in normality (Schmider et al. 2010) and the majority of normality and homogeneity of variance tests were nonsignificant (38 tests out of 44). Nonetheless, the results of groups that violated assumptions should be interpreted with caution, particularly TOC and chrysophyte density, which deviated the most from normal distributions.

**Table 1:** Two‐way ANOVA and variance partitioning results for each response variable. *N* = 24 for each test (4 replicates per treatment). Dinoflagellate, desmid, euglenoid, and synurophyte phytoplankton abundance and biovolume were not analyzed statistically due to low sample size but are included in total phytoplankton calculations. Significant *p* values (*α* = 0.05) are bolded with their respective *R* ^2^.

Response variable	*R* ^2^_light_	*R* ^2^_zoop_	*R* ^2^_light:zoop_	*p*_light_	*p*_zoop_	*p*_light:zoop_
Total phytoplankton abundance^*^	0.11	0.05	0.01	0.1492	0.6115	0.8543
Chlorophyte abundance^*^	0.01	0.01	0.08	0.7348	0.8949	0.4658
Diatom abundance^*^	**0.84**	**0.05**	0.00	**4.293 × 10** ^**−10**^	**0.0180**	0.9273
Cyanobacteria abundance^*^	0.00	0.05	0.07	0.7673	0.5994	0.5089
Cryptophyte abundance^*^	**0.33**	0.07	0.16	**0.0016**	0.2444	0.0558
Chrysophyte abundance^*^	**0.86**	**0.04**	0.00	**1.719 × 10** ^**−10**^	**0.0451**	0.7562
Haptophyte abundance^†^	0.01	0.01	0.02	0.7439	0.9545	0.8106
Total phytoplankton biovolume	**0.51**	0.10	0.01	**0.0001**	0.1366	0.8210
Chlorophyte biovolume^*^	0.17	0.11	0.02	0.0505	0.2695	0.7380
Diatom biovolume^*^	**0.71**	0.01	0.01	**2.173 × 10** ^**−6**^	0.7891	0.7443
Cyanobacteria biovolume^*^	0.02	0.07	0.09	0.5712	0.4618	0.3740
Cryptophyte biovolume^*^	**0.35**	**0.27**	0.09	**0.0002**	**0.0022**	0.0802
Chrysophyte biovolume^*^	**0.86**	0.04	0.00	**2.694 × 10** ^**−10**^	0.0505	0.6920
Haptophyte biovolume	0.03	0.03	0.03	0.4680	0.7551	0.7179
Rotifer abundance	0.03	0.03	0.02	0.4581	0.4174	0.5253
Rotifer biomass^*^	0.04	**0.34**	0.03	0.2605	**0.0173**	0.6789
TP	0.11	0.02	0.03	0.1291	0.5490	0.4007
SRP	**0.73**	**0.15**	0.00	**8.079 × 10** ^**−10**^	**9.489 × 10** ^**−5**^	0.6969
TOC	0.00	0.00	0.01	0.7872	0.8323	0.6004
TN	0.08	**0.20**	0.09	0.1350	**0.0211**	0.1080
Ammonium	**0.72**	**0.21**	0.00	**2.342 × 10** ^**−12**^	**8.690 × 10** ^**−8**^	0.9861
Nitrate	0.01	0.00	0.00	0.7133	0.8282	0.8996

^*^ Data were natural‐log transformed.

^†^ Data were square‐root‐transformed before ANOVA.

**Fig. 2:**
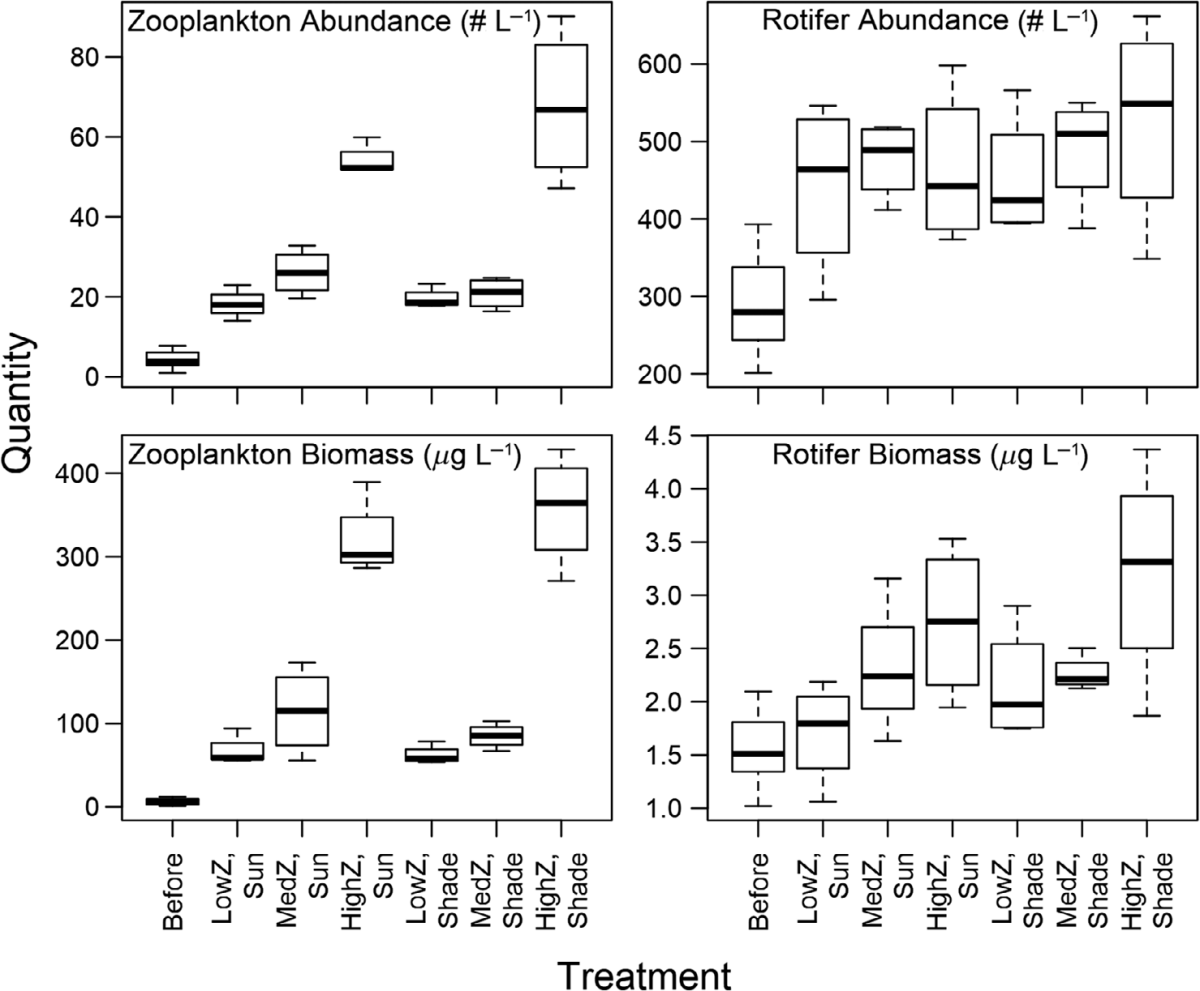
Crustacean zooplankton and rotifer abundance and biomass. “Before” indicates data collected from the water column of Shelburne Pond at the beginning of experiments. “Z” indicates zooplankton level. Center lines indicate medians, boxes indicate first and third quartiles of data, and whiskers indicate minimum and maximum of data.

Zooplankton abundance and biomass were significantly different between treatments (ANOVA; *F*_1,22_ = 225.1, *p* < 0.0001), but differences were not as great as intended (Fig. 2). Our low zooplankton treatments averaged (± SD) 64.5 ± 14.21 *μ*g dry L^−1^, medium zooplankton treatments averaged 99.9 ± 5.36 *μ*g dry L^−1^, and high zooplankton treatments averaged 338.6 ± 56.93 *μ*g dry L^−1^ (Table 2). That is, our intended “10x” zooplankton level had 3.4x the zooplankton biomass as our intended “1x” treatment. Consequently, we refer to zooplankton levels as low, medium, and high rather than 0x, 1x, and 10x. In all treatments, zooplankton biomass was dominated by *Diacyclops thomasi* and zooplankton abundance was dominated by both *D. thomasi* and copepod nauplii (Supporting Information Fig. S2). Crustacean zooplankton body size followed a bimodal distribution with a smaller peak that represented copepod nauplii and *Chydorus* spp. and the larger peak represented other adult zooplankton (Supporting Information Fig. S3). Treatments with high zooplankton density had a smaller relative proportion of smaller zooplankton (Supporting Information Fig. S3).

**Table 2:** Factor‐level means with 95% confidence intervals (in square brackets) for all response variables. Letters under zooplankton treatments (A or B) denote results of Tukey's test for pair‐wise comparisons between zooplankton levels when zooplankton was a significant factor in ANOVAs. Different letters denote significant differences between zooplankton levels. Absence of letters for a particular response variable indicates that the ANOVA was nonsignificant, and we did not perform Tukey tests.

Variable	Sun (*n* = 12)	Shade (*n* = 12)	Low zooplankton (*n* = 8)	Medium zooplankton (*n* = 8)	High zooplankton (*n* = 8)
Zooplankton abundance (# L^−1^)	32.80 [22.27, 43.33]	36.04 [19.82, 52.27]	18.89 [16.41, 21.38]	23.48 [19.04, 27.92]	60.89 [48.63, 73.16]
Zooplankton biomass (*μ*g L^−1^)	167.26 [90.59, 243.92]	168.08 [76.25, 259.91]	64.53 [52.63, 76.44]	99.90 [67.78, 132.02]	338.56 [290.97, 386.16]
Total phytoplankton abundance (cells mL^−1^)	8.10 × 10^3^ [6.34 × 10^3^, 9.85 × 10^3^]	6.41 × 10^3^ [4.15 × 10^3^, 8.67*10^3^]	8.11 × 10^3^ [4.43 × 10^3^, 1.18 × 10^4^]	7.58 × 10^3^ [5.35 × 10^3^, 9.81*10^3^]	6.07 × 10^3^ [4.16 × 10^3^, 7.97 × 10^3^]
Chlorophyte abundance (cells mL^−1^)	476.75 [162.11, 791.39]	537.04 [262.13, 811.95]	583.66 [93.45, 1.07 × 10^3^]	503.40 [144.63, 862.16]	433.63 [118.80, 748.46]
Diatom abundance (cells mL^−1^)	1361.27 [1051.16, 1671.38]	238.34 [166.64, 310.03]	958.84 [288.67, 1.63 × 10^3^] **A**	883.03 [303.96, 1.46 × 10^3^] **A**	557.54 [140.42, 974.66] **B**
Cyanobacteria abundance (cells mL^−1^)	3759.79 [2203.47, 5316.10]	4.13 × 10^3^ [2.05 × 10^3^, 6.20 × 10^3^]	4.41 × 10^3^ [937.83, 7.88 × 10^3^]	4.31 × 10^3^ [2.83 × 10^3^, 5.78 × 10^3^]	3.11 × 10^3^ [1.28 × 10^3^, 4.95 × 10^3^]
Cryptophyte abundance (cells mL^−1^)	196.12 [127.98, 264.25]	72.39 [39.08, 105.71]	161.83 [81.30, 242.37]	83.66 [44.69, 122.62]	157.28 [40.05, 274.50]
Chrysophyte abundance (cells mL^−1^)	988.20 [775.79, 1200.6]	162.35 [116.61, 208.10]	718.86 [200.24, 1.24*10^3^] **A**	527.49 [178.09, 876.90] **AB**	479.47 [132.56, 826.39] **B**
Haptophyte abundance (cells mL^−1^)	1283.44 [703.39, 1863.49]	1.25 × 10^3^ [950.62, 1.55 × 10^3^]	1.26 × 10^3^ [570.11, 1.94 × 10^3^]	1.24 × 10^3^ [894.75, 1.59 × 10^3^]	1.31 × 10^3^ [549.38, 2.06 × 10^3^]
Total phytoplankton biovolume (*μ*m^3^ mL^−1^)	5.80 × 10^5^ [4.82 × 10^5^, 6.78 × 10^5^]	3.05 × 10^5^ [2.24 × 10^5^, 3.86 × 10^3^]	5.15 × 10^5^ [3.24 × 10^5^, 7.05 × 10^5^]	4.44 × 10^5^ [2.85 × 10^5^, 6.03 × 10^5^]	3.68 × 10^5^ [2.31 × 10^5^, 5.06 × 10^5^]
Chlorophyte biovolume (*μ*m^3^ mL^−1^)	4.29 × 10^4^ [2.52 × 10^4^, 6.06 × 10^4^]	2.22 × 10^4^ [1.54 × 10^4^, 2.89 × 10^4^]	3.01 × 10^4^ [1.11 × 10^4^, 4.90 × 10^4^]	4.16 × 10^4^ [1.76 × 10^4^, 6.56 × 10^4^]	2.60 × 10^4^ [1.20 × 10^4^, 4.00 × 10^4^]
Diatom biovolume (*μ*m^3^ mL^−1^)	1.30 × 10^5^ [9.70 × 10^4^, 1.64 × 10^5^]	3.00 × 10^4^ [1.47 × 10^4^, 4.53 × 10^3^]	8.46 × 10^4^ [3.04 × 10^4^, 1.39 × 10^5^]	8.04 × 10^4^ [2.52 × 10^4^, 1.36 × 10^5^]	7.57 × 10^4^ [1.48 × 10^4^, 1.37 × 10^4^]
Cyanobacteria biovolume (*μ*m^3^ mL^−1^)	1.24 × 10^5^ [8.03 × 10^4^, 1.68 × 10^5^]	1.50 × 10^5^ [8.57 × 10^4^, 2.15 × 10^5^]	1.47 × 10^5^ [4.66 × 10^4^, 2.48 × 10^5^]	1.55 × 10^5^ [1.06 × 10^5^, 2.04 × 10^5^]	1.10 × 10^5^ [5.09 × 10^4^, 1.68 × 10^5^]
Cryptophyte biovolume (*μ*m^3^ mL^−1^)	5.63 × 10^4^ [3.24 × 10^4^, 8.02 × 10^4^]	2.18 × 10^4^ [3.92 × 10^3^, 3.97 × 10^4^]	6.84 × 10^4^ [3.28 × 10^4^, 1.04 × 10^5^] **A**	2.34 × 10^4^ [9.61 × 10^3^, 3.71 × 10^4^] **B**	2.54 × 10^4^ [49.46, 5.08 × 10^4^] **B**
Chrysophyte biovolume (*μ*m^3^ mL^−1^)	1.59 × 10^5^ [1.18 × 10^5^, 1.99 × 10^5^]	2.23 × 10^4^ [1.61 × 10^4^, 2.85 × 10^4^]	1.16 × 10^5^ [2.68 × 10^4^, 2.05 × 10^5^]	7.79 × 10^4^ [2.66 × 10^4^, 1.29 × 10^5^]	7.80 × 10^4^ [1.16 × 10^4^, 1.44 × 10^4^]
Haptophyte biovolume (*μ*m^3^ mL^−1^)	3.36 × 10^4^ [1.77 × 10^4^, 4.96 × 10^4^]	3.19 × 10^4^ [2.52 × 10^4^, 3.86 × 10^4^]	2.81 × 10^4^ [1.73 × 10^4^, 3.89 × 10^4^]	3.25 × 10^4^ [2.09 × 10^4^, 4.41 × 10^4^]	3.77 × 10^4^ [1.47 × 10^4^, 6.07 × 10^4^]
Rotifer abundance (# L^−1^)	461.19 [407.58, 514.80]	489.62 [428.92, 550.32]	447.33 [371.74, 522.93]	483.28 [435.24, 531.32]	495.60 [399.16, 592.04]
Rotifer biomass (*μ*g L^−1^)	2.26 [1.80, 2.71]	2.54 [2.04, 3.05]	1.93 [1.49, 2.37] **A**	2.29 [1.93, 2.65] **AB**	2.98 [2.26, 3.70] **B**
TP (*μ*g L^−1^)	40.80 [38.20, 43.41]	43.58 [40.84, 46.32]	41.47 [38.18, 44.75]	42.19 [37.29, 47.10]	42.91 [40.12, 45.70]
SRP (*μ*g L^−1^)	9.56 [8.35, 10.77]	15.50 [14.31, 16.70]	11.43 [8.84, 14.02] **A**	11.75 [8.64, 14.87] **A**	14.41 [11.51, 17.30] **B**
TOC	8.69 [8.47, 8.91]	8.66 [8.55, 8.77]	8.76 [8.45, 9.06]	8.60 [8.46, 8.74]	8.66 [8.46, 8.86]
TN (mg L^−1^)	0.76 [0.72, 0.79]	0.78 [0.76, 0.81]	0.76 [0.72, 0.80] **A**	0.755 [0.72, 0.79] **A**	0.80 [0.77, 0.83] **A**
Ammonium (mg L^−1^)	0.14 [0.13, 0.15]	0.18 [0.17, 0.19]	0.15 [0.13, 0.17] **A**	0.15 [0.13, 0.17] **A**	0.17 [0.16, 0.19] **B**
Nitrate (mg L^−1^)	0.11 [0.10, 0.12]	0.11 [0.10, 0.12]	0.11 [0.10, 0.12]	0.11 [0.10, 0.12]	0.11 [0.10, 0.12]

Phytoplankton samples comprised diatoms, chlorophytes, cyanobacteria, cryptophytes, dinoflagellates, chrysophytes, haptophytes, synurophytes, desmids, and euglenoids (Table 3).

**Table 3:** Phytoplankton genera found in mesocosms.

Taxonomic group	Genera	Taxonomic group	Genera
**Diatoms**	*Asterionella*	**Cyanobacteria**	*Aphanocapsa*
	*Diatoma*		*Dolichospermum*
	*Fragilaria*		*Microcystis*
	*Navicula*		*Pseudanabaena*
	*Stephanodiscus*		*Woronichinia*
**Chlorophytes**	*Ankistrodesmus*	**Cryptophytes**	*Chroomonas*
	*Chlamydomonas*		*Cryptomonas*
	*Crucigenia*		*Komma*
	*Dictyosphaerium*	**Dinoflagellates**	*Peridinium*
	*Eudorina*	**Chrysophytes**	*Chrysococcus*
	*Franceia*		*Dinobryon*
	*Gonium*	**Haptophytes**	*Chrysochromulina*
	*Kirchneriella*	**Synurophytes**	*Mallomonas*
	*Micractinium*	**Desmids**	*Cosmarium*
	*Oocystis*		*Staurastrum*
	*Scenedesmus*	**Euglenoids**	*Euglena*
	*Schroederia*		
	*Selenastrum*		
	*Tetrabaena*		
	*Tetraspora*		

**Fig. 3:**
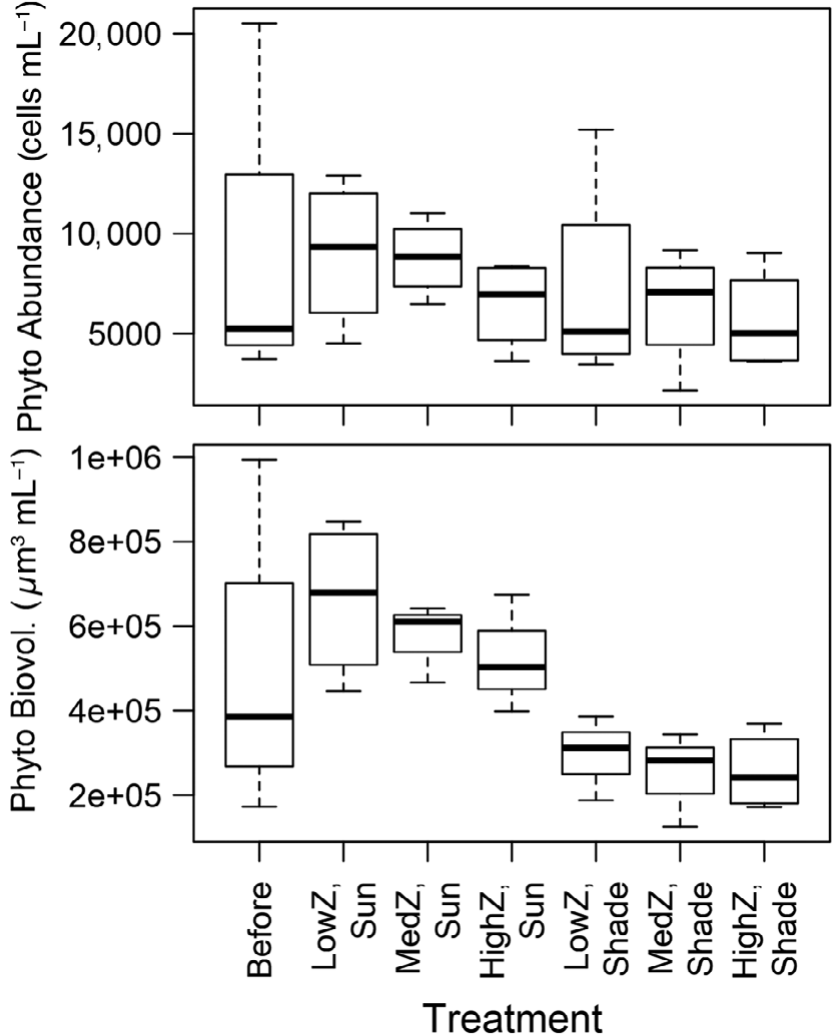
Total phytoplankton biovolume and abundance. “Before” indicates data collected from the water column of Shelburne pond at the beginning of experiments. “Z” indicates zooplankton level. Center lines indicate medians, boxes indicate first and third quartiles of data, and whiskers indicate minimum and maximum of data.

**Fig. 4:**
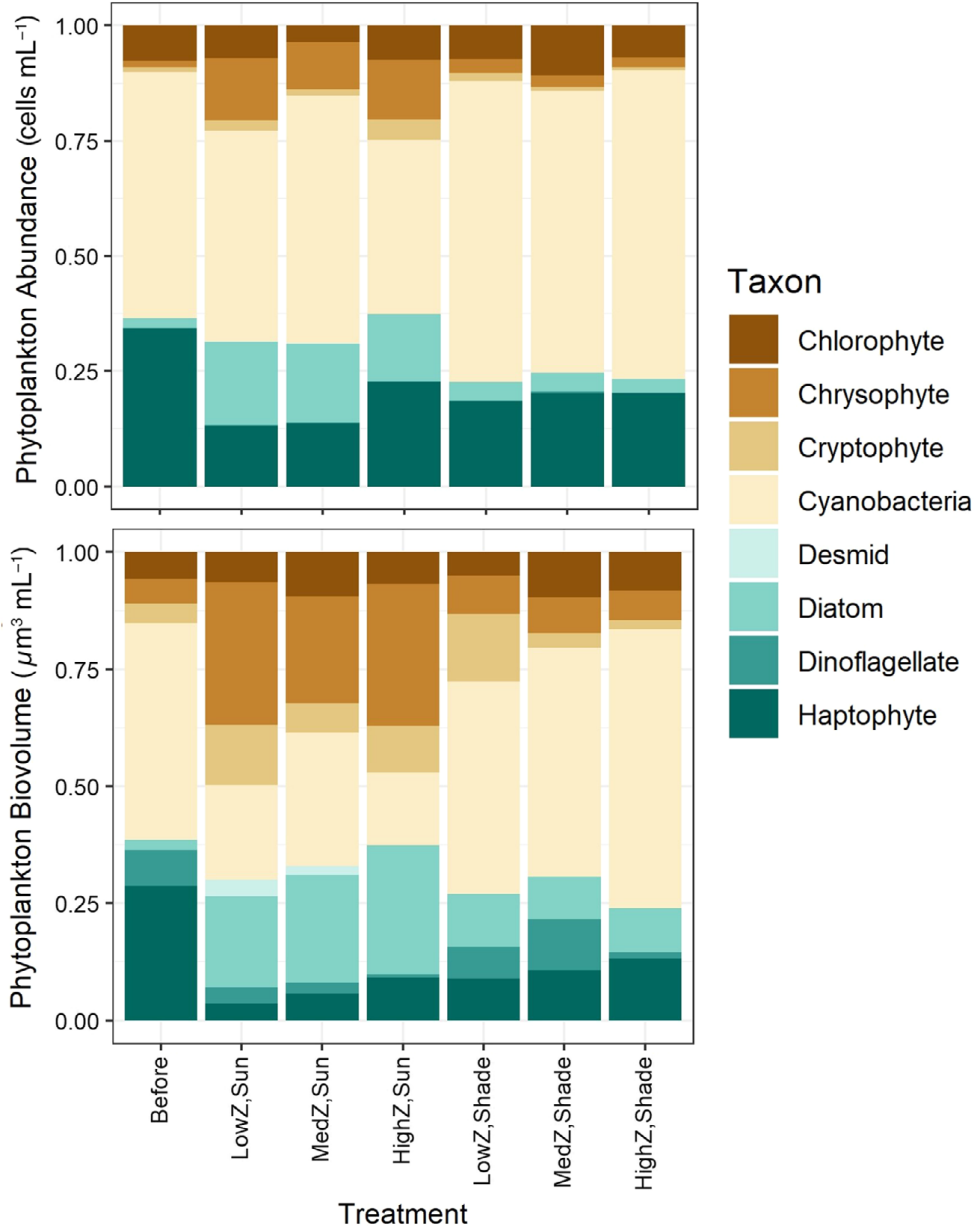
Phytoplankton proportional abundance and biovolume by taxonomic group. “Before” indicates data collected from the water column of Shelburne Pond at the beginning of experiments. “Z” indicates zooplankton level.

Differences in phytoplankton community composition among treatments were primarily driven by light, although diatoms, cryptophytes, and chrysophytes were also significantly affected by zooplankton levels. The biovolume of total phytoplankton and the abundance and biovolume of diatoms, cryptophytes, and chrysophytes were significantly higher with light (Tables 1, 2; Figs. 3, 4). Our light treatment most strongly affected diatoms (*R* ^2^_light_ = 0.84 for abundance and *R* ^2^_light_ = 0.71 for biovolume) and chrysophytes (*R* ^2^_light_ = 0.86 for abundance and *R* ^2^_light_ = 0.86 for biovolume) (Table 1). Diatom abundance, chrysophyte abundance, and cryptophyte biovolume were significantly lower with higher zooplankton treatments, but zooplankton explained less variation between factor levels than light treatments (*R* ^2^_zoop_ = 0.05 for diatom abundance, *R* ^2^_zoop_ = 0.04 for chrysophyte abundance, and *R* ^2^_zoop_ = 0.27 for cryptophyte biovolume) (Table 1). Tukey's tests showed that only the high zooplankton treatment had an effect on diatom abundance (Table 2), whereas chrysophyte abundance was different only at the lowest compared to the highest zooplankton level and cryptophyte biovolume was only significantly higher at the lowest zooplankton level (Table 2). Cyanobacteria and haptophytes were also found in all samples but did not vary by treatment (Table 1). No interaction terms for any response variables were significant in ANOVAs (Table 1).

**Fig. 5:**
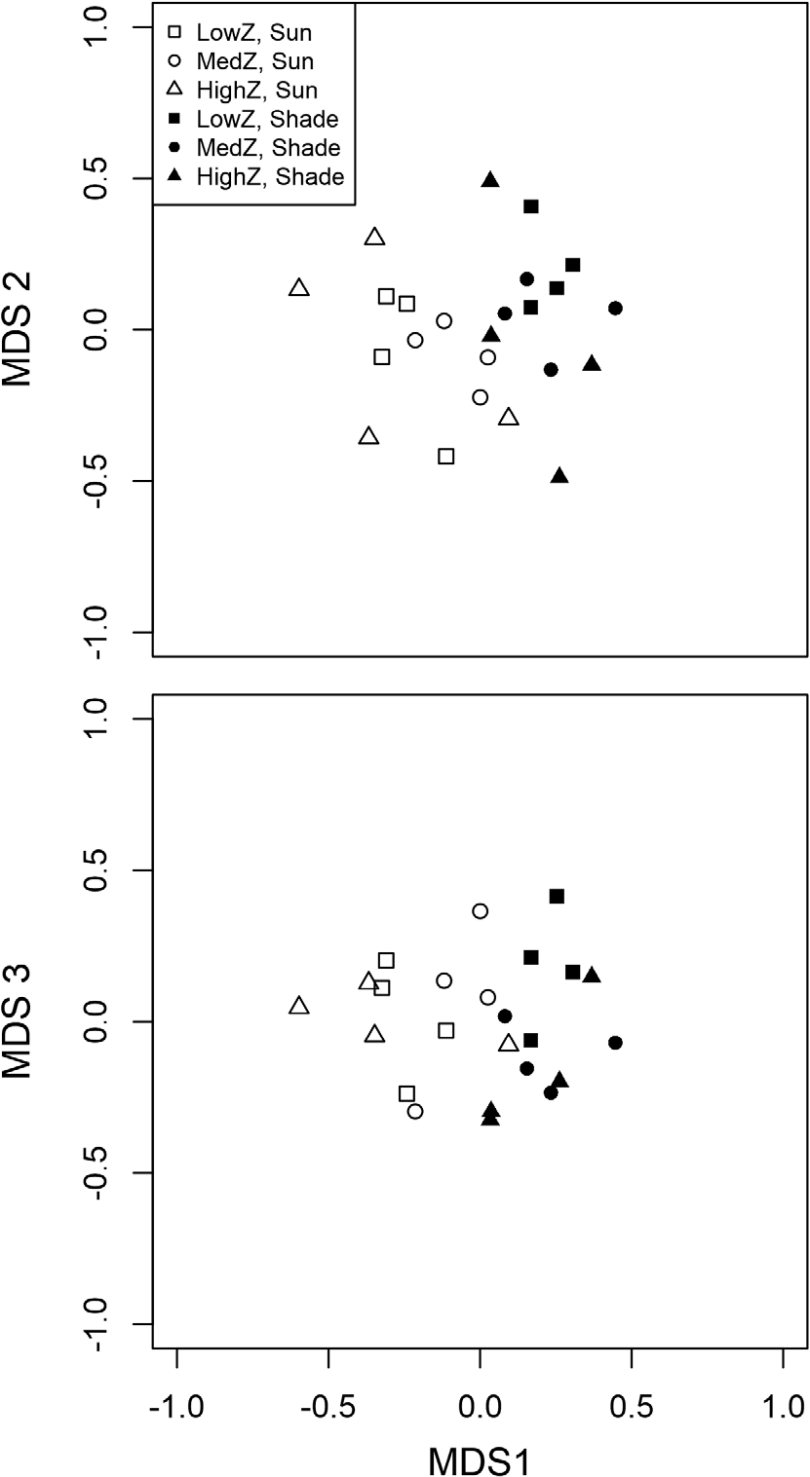
Results from nMDS analysis on phytoplankton community composition with Bray‐Curtis distance and three axes (stress = 0.15). Each point represents one replicate. The top panel represents the first two axes and the bottom panel represents the first and third axes.

Nonmetric multidimensional scaling showed a clear difference between shaded and unshaded phytoplankton communities for all axes, and only a slight difference due to the zooplankton treatment (Fig. 5). Treatments at the beginning of experiments were more similar to shaded treatments than unshaded treatments. Our final ordination had three axes to reduce stress from 0.22 with two axes to 0.15 with three axes (Fig. 5). Phytoplankton genera that drove separations along nMDS axes were mostly rare species that were only found in some treatments such as *Crucigenia*, *Diatoma*, *Aphanocapsa*, and *Dictyosphaerium* for the first axis and *Oocystis*, *Stephanodiscus*, *Selenastrum*, and *Gonium* for the second axis (Supporting Information Fig. S4). More common genera such as *Chrysochromulina* and *Woronichinia* were found across treatments, so contributed little to separations in nMDS axes (Supporting Information Fig. S4). perMANOVA indicated a significant effect of light treatment (pseudo *F*_1,20_ = 12.3; *p* = 0.001) but no effect of zooplankton treatment (pseudo *F*_2,20_ = 1.36; *p* = 0.184) on overall phytoplankton community composition.

Rotifer abundance was not dependent on experimental conditions, but rotifer biomass was significantly affected by zooplankton levels. Rotifer biomass was higher with higher zooplankton levels (Fig. 2; Table 1), and rotifer biomass was significantly higher for the pairwise comparison between the highest and lowest zooplankton levels (Table 2). Changes in zooplankton treatments accounted for 34% of the variation in rotifer biomass among treatments (i.e., *R* ^2^_zoop_ = 0.34). *Keratella cochlearis* was the dominant rotifer species found in mesocosms and made up > 90% of individuals, but only 39% of biomass due to their small size (Supporting Information Fig. S5). Other rotifers found in mesocosms were *Keratella hiemalis*, *Brachionus angularis*, *Asplanchna* spp., and *Filinia* spp.

**Fig. 6:**
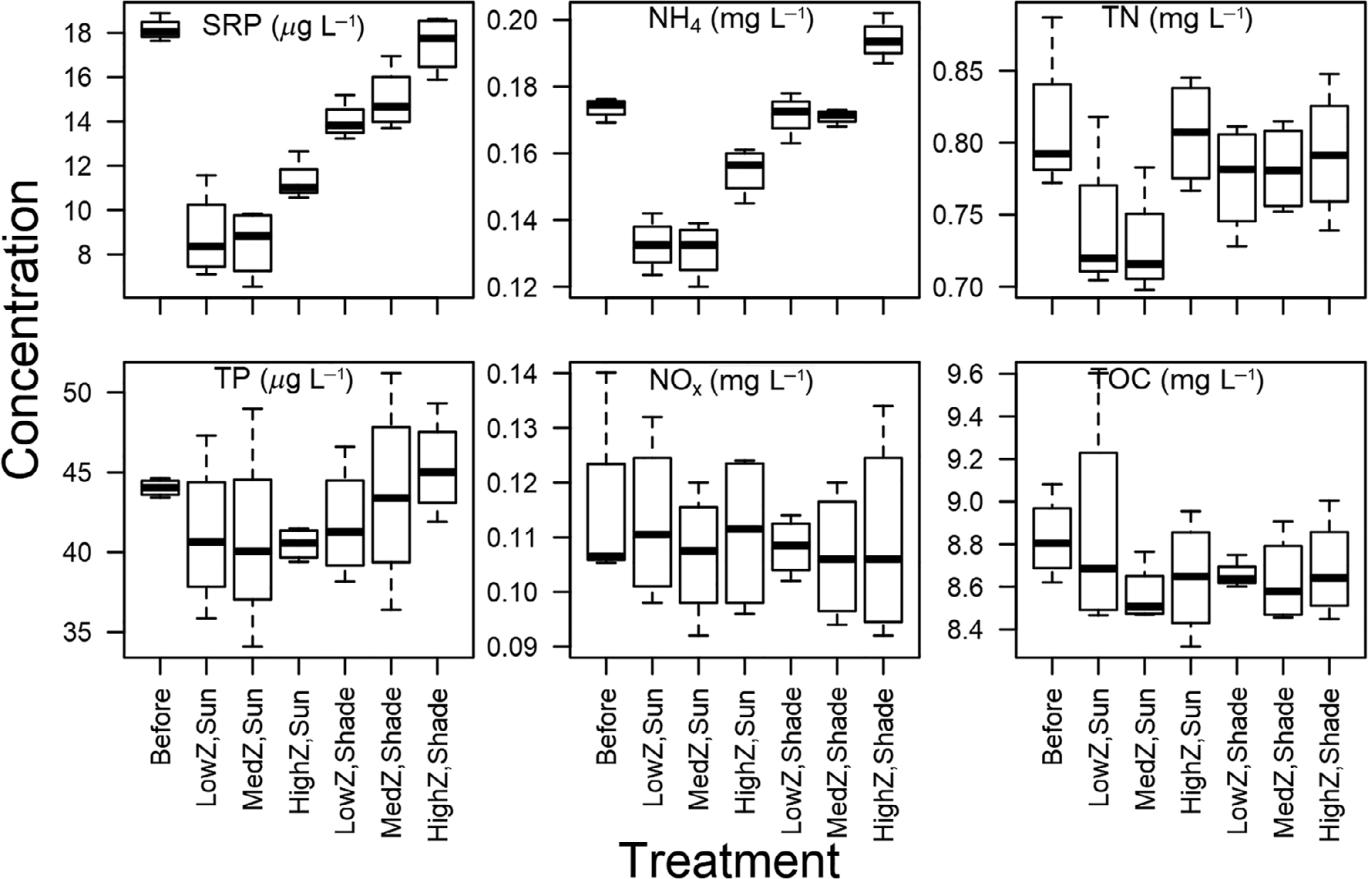
Nutrient results from experimental treatments. “Before” indicates data collected from the water column of Shelburne Pond at the beginning of experiments. “Z” indicates zooplankton level. Center lines indicate medians, boxes indicate first and third quartiles of data, and whiskers indicate minimum and maximum of data.

Some nutrient levels differed between treatments (Fig. 6; Table 1). SRP and ammonium were significantly lower in unshaded compared to shaded conditions and significantly higher at high zooplankton levels. Pairwise comparisons for SRP and ammonium showed significantly higher concentrations at high vs. low zooplankton and high vs. medium zooplankton, but not between medium and low zooplankton levels (Table 2). Light explained 73% of the variation in SRP and 72% of the variation in ammonium, while zooplankton explained 15% of the variation in SRP and 21% in ammonium between factor levels (Table 1). Zooplankton also increased TN and explained 20% of the variance between treatments (Table 1). However, all pairwise comparisons between zooplankton levels were nonsignificant and had similar factor‐level means (Table 2). Neither light nor zooplankton significantly affected TP, NO_x_, or TOC (Fig. 6; Table 1).

## Discussion

We found that light had a stronger influence on phytoplankton biovolume and community composition than zooplankton grazing in our under‐ice carboy experiment. The results support the hypothesis that bottom‐up control exceeds top‐down control on phytoplankton under ice. Specifically, light manipulation had the highest relative importance for chrysophytes and diatoms. However, zooplankton grazing had non‐negligible effects on some phytoplankton groups and on nutrient concentrations. The significance of zooplankton in our experiment contrasts with the PEG Model assumption that the effects of zooplankton grazing are low during winter (Sommer et al. 1986, 2012). Actively overwintering zooplankton have the potential to impact phytoplankton biovolume under ice, likely through selective feeding and nutrient cycling. Other studies have found higher winter abundances of large grazing zooplankton than in our highest treatment (Plew and Pennak 1949; Kasprzak et al. 2007), suggesting that our experimental levels are within a reasonable scope of zooplankton densities that occur under ice. Earlier literature suggests that winter zooplankton function primarily as a standing stock to graze the spring phytoplankton bloom after ice‐out (Sommer et al. 2012). Our results, however, show that the role of winter zooplankton may be more nuanced. Changes in phytoplankton community dynamics from zooplankton biomass together with light conditions could have direct consequences for which phytoplankton communities are present at ice‐out.

Difference in light availability had a stronger effect on phytoplankton community composition than difference in zooplankton levels, supporting the hypothesis that light is the main driver for the different dynamics of phytoplankton (biovolume, abundance, and composition). The difference was apparent in nMDS visualizations, perMANOVA of the entire phytoplankton community, and ANOVA analysis of specific phytoplankton groups. In particular, we observed higher proportion of chrysophytes (mostly *Chrysococcus*) and diatoms when more light was available. *Chrysococcus* can be successful under the ice in north temperate lakes (Phillips and Fawley 2002) so *Chrysococcus* as a dominant phytoplankton in Shelburne Pond during winter is not surprising. Although *Chrysococcus* is a known mixotroph that can withstand low light conditions (Olrik 1998), it still responded strongly to high light in our experiment. Diatoms are also often abundant under clear ice with high light transmission similar to our unshaded experimental conditions (Katz et al. 2015). Interestingly, the changes we observed in phytoplankton community composition took place over just 2 weeks, indicating that highly variable or rapidly changing light environments (e.g., patchy snow, rapid snowfall on top of clear ice, or snow that is abruptly windswept off ice) could have large impacts on phytoplankton community structure over relatively short time scales. The variation in light transmission in our study was well within the range of natural variation that would be expected during winter in a single lake (Bolsenga and Vanderploeg 1992).

Light treatments significantly altered ammonium and SRP presumably through increased phytoplankton production. Ammonium was lowest in treatments with high phytoplankton levels, but NO_x_ remained constant across treatments. These results suggest that SRP and ammonium were taken up for phytoplankton growth. SRP and ammonium most likely did not limit phytoplankton growth in mesocosms because concentrations of both were higher than concentrations found in Shelburne Pond during summer when phytoplankton production is highest (Ferber et al. 2004).

Despite discrepancies between expected and actual zooplankton biomass in our treatments, high zooplankton levels still had a major effect in carboys through consumption of certain phytoplankton groups and alteration of nutrient concentrations. Zooplankton significantly decreased total phytoplankton biovolume, cryptophyte biovolume, and diatom abundance, suggesting that zooplankton selectively grazed larger cryptophytes (specifically *Cryptomonas*) and smaller or non‐colonial diatoms. Diatoms and cryptophytes are known food sources for both rotifers and crustacean zooplankton (Mohr and Adrian 2002; Zhou et al. 2011; Tõnno et al. 2016). Furthermore, we found higher SRP and ammonium with high zooplankton levels, which is likely the result of zooplankton excretion (Oliver et al. 2015). TN showed significant effects of zooplankton in ANOVAs. Pairwise comparisons among zooplankton levels, however, were nonsignificant. Zooplankton effects were most evident in contrasts between high and low zooplankton levels and may have been stronger if we were able to manipulate the entire zooplankton community rather than just large‐bodied zooplankton. Lack of interaction effects between light and zooplankton levels indicates that our treatments were independent of one another and that zooplankton did not respond to different light treatments. Although top‐down control from zooplankton grazing was smaller relative to bottom‐up control from light limitation in this experiment, zooplankton appear to have the potential to indirectly affect phytoplankton though mobilization of nutrients during a period when external nutrient inputs are low.

Rotifers increased in biomass as crustacean zooplankton increased. We would expect rotifers to decrease when crustacean zooplankton are abundant because crustacean zooplankton typically outcompete rotifers when phytoplankton resources are limited (Fussmann 1996), and copepods such as *Diacyclops* may directly consume rotifers (Ciros‐Pérez et al. 2004). One possibility is that the increased nutrients from adding zooplankton increased primary production that was then consumed by zooplankton and rotifers. However, we cannot evaluate whether nutrient cycling and phytoplankton regeneration rates increased because we only measured standing stock of phytoplankton at the end of experiments. Another potential explanation is that additional rotifers were added with the zooplankton treatments. However, this possibility is unlikely because analysis of the zooplankton retained on 350‐*μ*m mesh sieve that was filtered into barrels indicated extremely low rotifer densities (0.26 individuals L^−1^). Another possibility is that large crustacean zooplankton outcompeted small crustacean zooplankton that may consume phytoplankton in the same size range as rotifers. In this case, a prevalence of large crustacean zooplankton would release rotifers from competition. This possibility seems most likely because small‐bodied zooplankton were found in lower proportions in treatments with more large‐bodied zooplankton.

Our mesocosm setup was effective in maintaining expected levels of physical parameters such as light and temperature but was less predictable in maintaining desired zooplankton levels. Water temperatures increased throughout the course of the experiment similarly among shaded and unshaded treatments, so temperature was not an influential factor in differences between treatments. The shade cloth covers maintained differences in light readings between treatments, including during a significant snowfall the night before we began extracting carboys (08 February). Zooplankton maintained differences between treatments, but at lower magnitudes than expected. The most likely explanation is that zooplankton experienced mortality as they were collected, sieved, and added to carboys. Alternatively, the highest zooplankton densities may have exceeded the carrying capacity of the carboys and experienced mortality during the experiment.

Mesocosm studies are necessarily limited in their scope to manipulate only the factors under study. In this experiment, we suspended our sealed carboys just below the ice. The limited mixing with the rest of the water column negated the potential for phytoplankton to settle to the bottom of the lake. However, our experiments were conducted during a period when the water was warming before ice‐out and was likely in a convective mixing state (Bruesewitz et al. 2015). Convective mixing could resuspend phytoplankton such as diatoms (Vehmaa and Salonen 2009), and thus, we would not expect phytoplankton to settle out as quickly as they would in a winter stratified state. Additionally, our sealed systems may have relaxed selective pressures for flagellated phytoplankton taxa by limiting their ability to migrate in the water column. Our 14‐d incubation period worked well to elucidate changes in phytoplankton communities between treatments; however, we caution others to consider the phytoplankton species present and their associated population growth rates to choose an appropriate incubation time for the system under study. We also excluded higher trophic levels, such as fish, which may actively forage during winter (Byström et al. 2006; Shuter et al. 2012; Block et al. 2020) and therefore affect trophic dynamics. Our limited number of light sensors did not allow us to determine the drivers of fluctuations in light under ice. For example, light in one replicate of the shaded treatment became lower compared to the unshaded treatment over time. Because our light tracking was limited to one carboy with a shade cloth cover and one without, we cannot determine whether this was caused by a biological phenomenon, such as fouling (which we did not observe), or by patchy snow. Despite these limitations, understanding planktonic food web interactions under ice in a controlled environment is an important first step to tease apart food web drivers under ice.

In this experiment, we demonstrated that the tested light levels were the more important driving factor on phytoplankton biovolume and community structure under ice compared to variation in the tested zooplankton levels using levels for both factors that would be expected under ice. Variations in light can also lead to significant changes in nutrient cycling. However, the role of zooplankton under ice should not be overlooked. Zooplankton appeared to decrease some phytoplankton taxa and altered nutrient concentrations in mesocosms, which suggests that we may miss important contributions of zooplankton in shaping phytoplankton communities and nutrient cycling under ice if we assume that overwintering zooplankton have negligible effects. Furthermore, high prevalence of copepod nauplii suggests that some crustacean zooplankton reproduced under ice. Winter copepod reproduction is often overlooked in temperate lakes, despite its occurrence in multiple systems (Vanderploeg et al. 1992; this study). Application of open‐water experimental techniques to ice‐covered ecosystems, such as under‐ice mesocosms, is an important step in disentangling food web drivers under ice.

Our results are relevant to understand what may happen with changing winter conditions associated with climate change. Ice cover duration of lakes is expected to shorten (Sharma et al. 2019), snowpack is expected to increase or decrease depending on the region of the world (Räisänen 2008), and most regions are predicted to have earlier and more protracted snowmelt (Klein et al. 2016; Musselman et al. 2017). Such changes are likely to affect phytoplankton community structure and productivity (Park et al. 2004; Huber et al. 2008), and if accompanied by changes in zooplankton abundance or community composition, could have important consequences for winter plankton community dynamics (e.g., Larsson and Wathne 2006; Wagner 2008) and the trajectory of plankton communities for the open‐water season (Preston and Rusak 2010; Feuchtmayr et al. 2012). Our experiment, which simulated a change in snow cover of just a few centimeters (Bolsenga and Vanderploeg 1992) for 2 weeks, was enough to significantly alter phytoplankton community structure. Consequently, seemingly minor events such as rain‐on‐snow events that melt a layer of snow, or slightly altered snowfall totals, may have disproportionately large effects on phytoplankton communities compared to the limited shading events that occur during the open‐water season. We also demonstrated two mechanisms by which zooplankton play a role in shaping under‐ice phytoplankton communities: selective grazing of some phytoplankton taxa, and alteration of nutrient cycling through excretion. Under‐ice experimental manipulation may provide an avenue to further disentangle the mechanisms that shape plankton communities under ice.

## Conflict of Interest

None declared.

## Supporting information


**Appendix S1**: Supporting information
